# Edible Insects as Human Food: Perceptions of Individuals from Six Countries

**DOI:** 10.3390/insects17040434

**Published:** 2026-04-17

**Authors:** Raquel P. F. Guiné, Sofia G. Florença, Anayansi Escalante-Aburto, Rosa María Mariscal-Moreno, César Ozuna, Lucio Rodríguez-Sifuentes, Cristina Chuck-Hernández, Marijana Matek Sarić, Nada M. Boustani, Elena Bartkiene, Cristina Filip, Simona Pârvu, Monica Tarcea

**Affiliations:** 1 CERNAS-IPV Research Centre, Polytechnic University of Viseu, 3504-510 Viseu, Portugal; sofiaflorenca@outlook.com; 2 Tecnologico de Monterrey, School of Engineering and Sciences, Monterrey 64849, Mexico; anayansi.escalante@tec.mx; 3 Tecnologico de Monterrey, The Institute for Obesity Research, Monterrey 64849, Mexico; cristina.chuck@tec.mx; 4 Departamento de Salud, Universidad Iberoamericana, Ciudad de Mexico 01219, Mexico; rosa.mariscal@ibero.mx; 5 Department of Food Science, Division of Life Sciences, University of Guanajuato, Irapuato 36500, Mexico; cesar.ozuna@ugto.mx; 6 Laboratorio de Biotecnología e Innovación, Facultad de Ciencias Biológicas, Universidad Autónoma de Coahuila, Torreón 25280, Mexico; lucio.rodriguez@uadec.edu.mx; 7 Department of Health Studies, University of Zadar, 23000 Zadar, Croatia; marsaric@unizd.hr; 8 Faculty of Business and Administration, Saint Joseph University, Beirut 1104 2020, Lebanon; nada.mallahboustany@usj.edu.lb; 9 Department of Food Safety and Quality, Lithuanian University of Health Sciences, LT-47181 Kaunas, Lithuania; elena.bartkiene@lsmuni.lt; 10 Department of Biochemistry and Environmental Chemistry, George Emil Palade University of Medicine, Pharmacy, Science, and Technology of Targu Mures, 540139 Targu Mures, Romania; cristina.filip@umfst.ro; 11 Department of Hygiene, Faculty of Medicine, Carol Davila University of Medicine and Pharmacy, 37 Dionisie Lupu, 020021 Bucharest, Romania; simona.parvu@umfcd.ro; 12 Department of Community Nutrition and Food Safety, George Emil Palade University of Medicine, Pharmacy, Science, and Technology of Targu Mures, 540139 Targu Mures, Romania; monica.tarcea@umfst.ro

**Keywords:** sociodemographic groups, factor analysis, cluster analysis, word analysis, eating insects

## Abstract

Edible insects (EIs) have been suggested as a sustainable alternative to meat from traditional sources. However, despite EIs being consumed by humans since time immemorial, it is also true that, in many areas, especially in Western countries, there is some resistance and even neophobia to their acceptance as food. This work aimed to investigate the perceptions about EIs and validate a corresponding scale. This descriptive cross-sectional study was carried out by means of a questionnaire survey on a sample of 3711 participants in six countries (Croatia, Lebanon, Lithuania, Mexico, Portugal and Romania). Data were analysed by factor and cluster analyses, as well as other statistical tools. The results showed that, out of the initial 36 items in the questionnaire, 24 were validated in the scale of perceptions, distributed across six factors. Cluster analysis showed that the participants could be grouped according to three clusters (Traditionalists, Shoppers and Innovators). Statistical tests revealed significant differences (*p* < 0.05) between the clusters in practically all sociodemographic and consumption variables studied. Finally, a word analysis of the 2263 words indicated by the participants when thinking about EIs revealed that the most frequent words were ‘disgusting’, ‘protein’, and ‘nutritive’ in all three clusters. However, differences were observed after the fourth position in the list of most frequent words. In conclusion, these results indicate that perceptions of EIs were highly variable across countries of origin and other sociodemographic groups, and that, globally, participants exhibited some neophobia towards EIs. However, they recognised their value in terms of nutrition, particularly protein. This work evidences some relevant aspects that can be useful to adapt and define targeted policies to demystify the negative perceptions about EIs and improve consumer acceptance, thus contributing to more sustainable food systems.

## Simple Summary

Consuming insects is traditional in some areas of the globe, while being considered very strange in most Western countries. In this study, we investigated the perceptions about edible insects (EIs) and how these varied across cultural groups. The results showed important differences between the participants from different countries, belonging to different sociodemographic groups, such as sex, education, income, or living environment. These results confirm the great influence of cultural and geographical factors on the perceptions towards EIs.

## 1. Introduction

The growth of the global population leads to significant changes; one of the most discussed and researched directions is the need for sustainable sources of protein. Thus, the current research focuses on finding sustainable ways to meet the growing population’s need for essential amino acids. The directions of research are diverse, ranging from plant-based meat to cultivated meat, and edible insects (EIs) are also of increasing importance [1].

Due to the adverse effects of animal husbandry, including land use occupation, greenhouse gas emissions, and high water consumption, there is pressure on traditional meat production systems to reduce their impact. In contrast, insect farming has a positive impact on the ecosystem [2]. Producing protein from insects requires between 21.20 and 99.60 MJ/kg of energy, generates greenhouse gas emissions of 15.10 kg of CO_2_, uses approximately 0.43 m^3^ of water, and occupies about 3.56 m^2^ of land, which are much more acceptable values than in the case of traditional animal husbandry [3]. Insect farming is also encouraged by the FAO as a sustainable ecological source of protein [4]. The consumption of insects as a staple food is a novelty for developed countries, mainly those in Europe. Still, in other parts of the world, such as Asia, Africa and Latin America, it is part of the traditional diet [5].

The level of acceptance of EIs is low in Europe compared to other regions. Globally, 2205 insect species have been identified for consumption in 128 countries, with the most diverse consumption being in Mexico, where 450 edible species have been identified [5]. In contrast, in Europe, only four insect species are accepted for human consumption: the yellow mealworm (Tenebrio molitor larva), the migratory locust (Locusta migratoria), the house cricket (Acheta domesticus), and the buffalo worm (Alphitobius diaperinus larva) [6]. The low acceptance of EIs is closely linked to psychological, cultural, and economic barriers. Their rejection was associated with keywords such as disgust, ignorance, social taboo, and the population’s living standards [5,7,8].

Global data suggest that the acceptance of insects as food is influenced by the countries’ level of economic development. Omuse et al. [5] demonstrated that the number of insect species consumed exhibits a negative association with GDP/capita, with countries having high GDP showing lower consumption. In comparison, countries with low or medium GDP have higher consumption of insect species. Similar results are supported by the FAO report (2013), which highlights that insects are frequently included in the diet in Latin America, Africa, and Asia, while in Europe and North America, the regions with high living standards, entomophagy is generally rejected and associated with poor hygiene [9].

Although there are many studies on the acceptance of EIs [10,11,12,13,14,15], there is still a clear need for research that explores consumer perceptions and behaviours in detail, particularly in demographically varied contexts, with a more in-depth examination of consumer behaviour and specific populations. Future research in this area should focus on ways to modify consumer perceptions of new food acceptance using validated instruments such as psychometric scales [16]. It is also relevant to segment consumers into clusters to help marketing campaign information reach target consumers more easily, through personalised information campaigns [17].

There are various works conducted on different samples exploring the consumption of EIs, focusing on different perspectives. The work by Ribeiro et al. [18] utilised emojis to register the emotional response of Portuguese consumers towards EIs. Nikbin et al. [19] studied the motivations of Chinese consumers to frequent a restaurant that serves meals containing EIs. Most of the studies focus on consumer behaviour and only a few focus on perceptions of consumers, and even in those cases, they are very limited to one country, for example, Benin [20], Belgium [21], or Germany [22]. There is a lack of knowledge on perceptions about EIs in comparison studies conducted across multiple countries. The objective of this study was to assess the perceptions about EIs in a multi-country approach. We intended to include countries covering a diversity of geographical locations, including the Iberian Peninsula (Portugal), Northern Europe (Lithuania), the South European Mediterranean Coast (Croatia), Central Europe (Romania), the Middle East Mediterranean Coast (Lebanon), and Latin America (Mexico), since this last country has some tradition of entomophagy, thus allowing to compare with other regions around Europe, where this tradition is not typically present. Underlying the main objective, another practical objective was to validate a corresponding scale, composed of several items focusing on diverse aspects related to the consumption of insects. The domains included in the scale included items about culture and tradition, items about gastronomic innovation and gourmet culinary, items about economic and social aspects, items about commercialisation and marketing, and items about health effects. Considering that sociodemographic and geographical variables can influence participants’ perceptions, this study was conducted in six countries with diverse regional influences.

## 2. Materials and Methods

### 2.1. Instruments and Data Collection

This study was based on a questionnaire survey conducted within the framework of the EISuFood project, previously validated by [23], to focus specifically on the items designed to measure perceptions, rather than objective knowledge. The items used were 36, as indicated in Table 1, and the participants manifested their agreement using a central five-point Likert scale (1 = strongly disagree, 2 = disagree, 3 = neither agree nor disagree, 4 = agree, 5 = strongly agree) [24].

The questionnaire also contained questions about consumption of EIs, namely ‘Have you ever eaten insects as culinary preparations, as snacks or other derived products?’, If you have never eaten insects, would you consider eating them?’, ‘In which circumstances would you consume?’, and ‘If you consume EIs, how often do you eat insects as culinary preparations, as snacks, or other derived products?’ All these questions were provided with pre-defined answering options.

Finally, the participants had the opportunity to express themselves by writing down up to five words that came to mind when hearing about EIs. In this case, it was only used for analysing the first word recorded by each participant who chose to answer this question.

**Figure 1:**
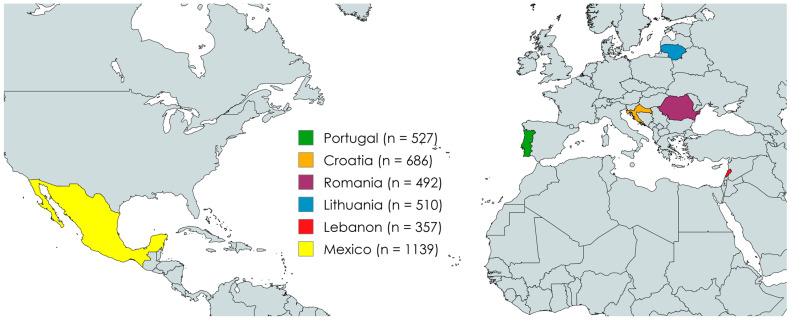
Geographical distributions of the countries included in this study.

This is a descriptive cross-sectional study, which was undertaken on a non-probabilistic sample of 3711 participants from six countries located in different regions of the globe and that have variable social and cultural backgrounds: Mexico (Latin America), Portugal (Iberian Peninsula), Lithuania (Northern Europe), Croatia (South European Mediterranean Coast), Romania (Central Europe) and Lebanon (Middle East Mediterranean Coast) (Figure 1).

To apply the questionnaire in the different countries, it was translated from the English version into the corresponding native language following a standard back-translation procedure. This research adhered to all ethical principles, including those outlined in the Declaration of Helsinki, and was approved by the Ethics Committee of the Polytechnic University of Viseu (reference number: 45/SUB/2021, dated 25 May 2021). Due to the geographical distribution of the countries, data were collected online during the year 2022 using Google Forms, exclusively from adult participants who provided informed consent, and recruitment followed a snowball sampling methodology. Although this procedure is not probabilistic, it has proven more effective when compared to multisite data collection [25].

### 2.2. Statistical Analyses

SPSS Version 29.0.1.0 from IBM, Inc. (Armonk, NY, USA) was used to perform all statistical analyses. Basic descriptive statistics were used, complemented with more complex analyses, specifically factor analysis (FA) and cluster analysis (CA).

In the first phase, exploratory FA was performed, using the method of Principal Component Analysis (PCA), to investigate if there was a grouping structure between the items. In the second phase, CA was performed over the factors obtained in the first step.

Before performing FA, the data were analysed to verify if they satisfied the following conditions to apply FA [26]:

(a) The correlation matrix was analysed to identify possible correlations between the variables.
(b) The Kaiser–Meyer–Olkin (KMO) measure of sample adequacy was calculated.
(c) Bartlett’s test was performed to evaluate correlations between variables.

The reference values considered to interpret the values of KMO were excellent for 0.9 ≤ KMO ≤ 1.0, good for 0.8 ≤ KMO < 0.9, acceptable for 0.7 ≤ KMO < 0.8, tolerable for 0.6 ≤ KMO < 0.7, bad for 0.5 ≤ KMO < 0.6, and unacceptable for KMO < 0.5 [27].

Once the adequacy of the data for applying FA was established, this was performed by considering the extraction method using PCA, with Varimax rotation and Kaiser Normalisation. Specifically, the number of factors extracted was determined by the condition that the eigenvalues were greater than one. Factor loadings with an absolute value lower than 0.4 were excluded, eliminating variables with inputs lower than 16% [28,29]. To analyse the internal consistency within each factor, Cronbach’s alpha (α) [26,30] was used. The reference values for alpha vary according to different authors, but values above 0.7 are generally considered good, with values above 0.8 being considered very good. Nevertheless, some authors also consider values over 0.5 acceptable [31,32,33]. When testing the internal consistency of each factor, besides calculating the value of alpha including all items in each factor, it was further tested whether the removal of any of the items would improve the internal consistency. If that happened, then the factor would be considered with only the items that allowed a higher internal consistency. For the cases where the removal of an item did not improve alpha, the factor was retained with all items if the value of alpha was acceptable, or the whole factor was removed in the case of unacceptable internal consistency. For a factor with only two items, this procedure is not viable since the removal of one item leaves a single item, and in that case, there is no possibility to assess internal reliability between items.

CA was applied to the factors defined in the previous stage by FA (six variables), and the process began by applying a series of hierarchical methods to determine the most suitable number of clusters. The six methods tested were: Between-Groups Linkage (BGL), Within-Groups Linkage (WGL), Nearest Neighbour (NN), Furthest Neighbour (FN), Centroid (CE), and Ward (WA). In all cases, the squared Euclidean distance method was used to measure the intervals. Based on the coefficients of the agglomeration schedule, the optimal number of groups to be formed was fixed at three.

In the next phase, the six methods were applied to the same variables, fixing three clusters. The solutions thus obtained were compared using contingency tables (crosstabs) to investigate possible stability (Table 2). Some of the solutions showed a possible similarity of about 100% (BGL, NN, CE). These results indicate a possible stability. Therefore, these three grouping solutions were used as the initial solutions for the next phase of the analysis, in which the partitive method of k-means was employed, as it is generally recommended and frequently used in clustering calculations [34].

The Chi-square test was used to identify potential differences between clusters in relation to the sociodemographic, geographic, and professional characteristics of the participants. A level of significance of 5% was used in the tests. Cramer’s V coefficients were used to investigate the strength of the associations between categorical variables. This coefficient varies in the range from 0 to 1, so that for V ≈ 0.1, the association is considered weak; for V ≈ 0.3, the association is considered moderate; and for V ≈ 0.5 or higher, the association is considered strong [35].

For the word analysis, the responses were simplified by grouping singulars/plurals and nouns/verbs to make the content consistent and avoid repeated concepts like ‘curious’ and ‘curiosity’ or ‘rich in protein’, ‘good protein’, and ‘high protein’, all unified to ‘protein’. Finally, certain concepts required simplification (e.g., ‘sustainable food’ to ‘sustainable’). Also, typographical errors were corrected to ensure the correctness of the words included in the analysis. Words or expressions without meaning were excluded (ex. ‘Hu…’, ‘Nop’, ’awh!’). To obtain the word clouds, the freeware software Free Word Cloud Generator was used (available online at: https://www.freewordcloudgenerator.com/generatewordcloud (accessed on 12 December 2025)).

### 2.3. Sample Characterisation

Table 3 presents the distribution of participants by the sociodemographic variables considered: age, gender, education, country, and living environment. Most participants were aged between 18 and 30 years (50.0%), with a smaller proportion of older participants (14.1% aged over 51 years). There were slightly more female participants than male (57.0% versus 42.2%), but the discrepancy was not so high as reported in similar studies [36,37,38].

The percentage of participants who had under-university studies was identical to those who completed a university degree, around 40% in both cases, and there were still 23.2% of participants who had postgraduate studies (master’s or doctoral degree).

Regarding the distribution by country, there was a predominance of Mexican participants (30.7%), and Lebanon was the least represented (9.6%), which is relatively in agreement with the relative size of the countries involved. Concerning the living environment, about two-thirds of the sample resided in an urban environment (68.8%).

Finally, with respect to family income, most participants declared to have an income equal to the average in their country (36.8%), while a significant part also had income above the average (30.2%).

Regarding the distribution of the participants by sociodemographic groups in the six studied countries, significant differences were observed in all cases (*p* < 0.001), and the associations were moderate to strong, with the values of Cramer’s V varying from a minimum of 0.195 for variable income to a maximum of 0.335 for variable education level.

## 3. Results

### 3.1. Factor Analysis

Before applying FA, it was verified whether the data were suitable for this type of statistical analysis. The results from the correlation matrix highlighted some significant correlations between the variables, with 12 values equal to or greater than 0.5. The highest correlation found in the matrix was 0.630, expressing the interaction between items 13 and 14. Also, it was confirmed that Bartlett’s test was significant (*p* < 0.001), leading to rejecting the null hypothesis ‘H0: The correlation matrix is equal to the identity matrix’. The KMO value was 0.920, which can be interpreted as excellent, based on the classification of Kaiser and Rice [27]. All these observations confirmed the suitability of the data for applying PCA and FA techniques. The anti-image matrix showed that all values of MSA (Measure of Sampling Adequacy) were higher than 0.5, meaning that all the variables were properly included in the analysis (there were only two values in the range 0.6–0.7, also two in the range 0.7–0.8, five values in the range 0.8–0.9, and 27 values were higher than 0.9).

The FA solution converged in 15 iterations, and the total variance explained by the solution obtained by FA with PCA and Varimax rotation was 52.50%, achieved through eight factors, with eigenvalues ranging from 8.25 for factor F1 to 1.05 for factor F8. The percentage of variance explained by each factor individually is presented in Table 4, which also summarises the items within each factor and their corresponding loadings. The item with the highest loading was item 14 in factor F1, and three items from the 36 initially considered were excluded because they had loadings under 0.4 (items 31. ‘Insects are used by some people in traditional medicine’, 33. ‘Industrial processed insect products are hygienic and safe’, and 36. ‘In certain countries, insects are approved officially for therapeutic treatment’).

Cronbach’s alpha (α) values were calculated to measure the internal consistency within each of the eight factors extracted by the FA solution [26]. The value of Cronbach’s alpha for factor F1 was 0.846, but it could be higher if item 10 were removed, increasing alpha to 0.858, which is considered very good [31,32,33]. Factors F2 and F3 have values of alpha that are considered good [31,32,33] (0.780 and 0.738, respectively), while factors F4 and F5 have alpha values that are acceptable [31,32,33] (0.695 and 0.678, respectively). With respect to factor F7, the value of alpha was unacceptable (α = 0.095), but this was increased to an acceptable value (α = 0.577) if items 1 and 24 were removed. Finally, factors F6 and F8 had values of alpha that were considered unacceptable, i.e., below 0.5 [31,32,33], and therefore should be excluded. As a result of the internal reliability analysis, several items were found unsuitable to be included in the analysis: 1. ‘Insects are considered a traditional food in my country’, 9. ‘Insects are considered exotic food’, 10. ‘Insects are traded as treats/delicacies’, 11. ‘Insects are associated with taboos and food neophobia (not wanting to eat unfamiliar foods)’, 19. ‘The market of edible insects is expected to decline in the future’, 24. ‘Edible insects are easy to find on sale in supermarkets’, 30. ‘Insect consumption is independent of marketing campaigns’, and 35. ‘Insects collected from the wild may be contaminated with pesticide residues’.

Considering these results, we conclude that the scale would be stronger if eleven items were removed [28] (items 1, 9, 10, 11, 19, 24, 30, 31, 33, 35, 36), as discussed earlier. Therefore, a final factor solution was run considering only 25 items instead of the 36 originally tested. For this group of items, the rotation converged in eight iterations, with an excellent value of KMO (KMO = 0.908) and a significant Bartlett’s test (*p* < 0.001). The final solution is presented in Table 5 and explains 56.14% of the variance, comprising six factors. Item 2. ‘Consuming insects is characteristic of developing countries’ was not included in any of the factors because of loading values under the established threshold of 0.4.

The first factor in Table 5 was named Gastronomy (GA) because its five items relate to gastronomy and culinary applications of EIs. Factor F2, which also includes five items, relates to social aspects of EIs, their contribution to livelihood and income, and food security of more vulnerable people, and therefore was named Social Aspects and Income (SI). Factor F3, with six items, gathered aspects related to the tradition and cultural aspects of EIs, and was therefore named Tradition (TR). Factor F4, with four items, refers to determinants of insect consumption and was named Motivations (MT). Factor F5 contained two items, both related to risks for human health, and it was named Health Risks (HR). Finally, factor F6, also with two items, was related to the commercialisation of EIs, and it was named Purchasing (PC). All factors had values of alpha higher than 0.5, being the internal consistency acceptable for factors F4, F4, and F6 (alpha equal to 0.695, 0.678, and 0.577, respectively), good for factors F2 and F3 (alpha equal to 0.780 and 0.724, respectively) and very good for factor F1 (α = 0.856) [31,32,33]. In all six factors, the values of alpha would not improve with the removal of any item, so the solution presented in Table 5 is considered the final solution, consisting of 24 items distributed by six factors.

In general, the item loadings for all factors were high, indicating a substantial contribution of the various items to the definition of each factor. Items with the highest loadings were item 14 (loading of 0.815 into factor F1) and item 34 (loading of 0.801 into factor F5), indicating that these items were those most strongly associated with the corresponding factors: item ‘Insects are recommended by some recognized chefs’ for factor Gastronomy (GS) and item ‘Insects and insect based foods are often infected by pathogens and parasites’ for factor Health Risks (HR).

### 3.2. Cluster Analysis

Table 6 presents the results obtained with the k-means clustering method using three initial solutions, which were generated from hierarchical methods (BGL, CE, and NN). The results showed relatively good convergence, both based on the percentage of cases in each of the three clusters and the proximity of the cluster centres, especially for initial solutions BGL and CE. The results of the ANOVA tests were significant in all cases (*p* < 0.001). They had high values of the test statistic (F), indicating a high degree of similarity within clusters and differentiation between clusters. It was observed that factors F1 (GA) and F3 (TR) contributed more intensively to the definition of the clusters (with higher values of F). In comparison, factor F2 (SI) contributed the least (with lower values of F). Based on the results, the final solution is accepted as that originating from the CE initial solution, and the results in this clustering structure are as follows:

- Cluster 1: Traditionalists—the strongest input from factor F3 linked with the tradition of EIs (includes 43% of the participants);
- Cluster 2: Shoppers—the strongest input from factor F6 linked with sales of EIs (includes 29% of the participants);
- Cluster 3: Innovators—the strongest negative input from factor F3 linked with tradition and positive input from factor F1 linked with gastronomy and innovative culinary applications (includes 28% of the participants).

### 3.3. Characterisation of the Clusters

Based on the cluster membership encountered in cluster analysis, Table 7 presents the distributions of the individuals in each cluster according to several sociodemographic characteristics. The results indicate some significant differences according to sex (*p* = 0.017), age (*p* < 0.001), education (*p* = 0.003), country (*p* < 0.001), and family income (*p* < 0.001), while for the living environment, no significant differences were observed. Regarding the values of Cramer’s coefficients, they were low in most cases, indicating a weak association between variables, except for country, in which the association was moderate (V = 0.290). In addition, significant differences were found for professionals in areas such as Agriculture, Biology, Health, and other professions, but again with weak associations (very low values of V).

Table 7 also shows that members of cluster C1 (Traditionalists) are predominant, and there are no distinguishing elements in terms of the variables sex, age, education, living environment, income, or professional area. Still, this cluster stands out for having a higher percentage of members from Lebanon, Mexico, and Romania. Regarding cluster C2 (Shoppers), it accounts for a higher percentage of participants with a lower education level (without completing a university degree), more participants from Portugal and Croatia, and a higher proportion of participants in professional areas such as Agriculture and Tourism. Finally, cluster C3 (Innovators) comprises more mature adults (aged between 31 and 50 years), with a higher proportion of participants from Lithuania, and a focus on areas such as Agriculture, Food/Nutrition, and Tourism.

Table 8 presents the results for cluster membership in relation to certain variables associated with the consumption of EIs. The results indicate that, again, the members of cluster C1 (Traditionalists) include participants from all groups in higher percentages, with a predominance of those who have already consumed EIs, who are willing to consume EIs whole or as ingredients in food preparations, and who consume EIs about once per week. Regarding cluster C2 (Shoppers), it includes a higher percentage of participants who would consider eating EIs in the event of food scarcity or to help preserve the planet, as well as participants who consume EIs about twice a week. Finally, cluster C3 (Innovators) includes more participants who consider eating insects to help preserve the planet and consume EIs very rarely, only about once a year.

### 3.4. Word Analysis According to the Clusters

**Figure 2:**
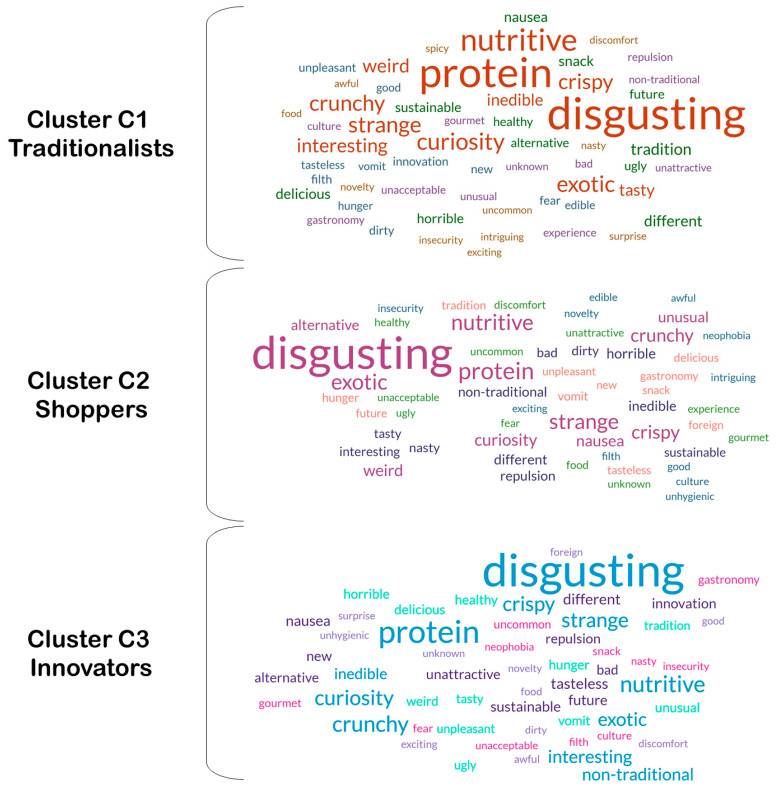
Word clouds for participants from the three clusters.

The 3702 participants expressed their perceptions of EIs through an open question asking them to associate words with EIs. From the word analysis performed, a total of 2263 words were obtained. For cluster C1, 882 words were obtained, of which 56 were unrepeated; in cluster C2, 711 words were obtained, of which 56 were unrepeated; and in cluster C3, 670 words were obtained, of which 55 were unrepeated (Complete Table in Appendix A). Figure 2 presents the word clouds obtained with the words corresponding to each of the clusters, i.e., given by participants who were classified in those clusters. As a general overview, it is noted that the most frequent word in all clusters was ‘disgusting’, regardless of the cluster. Also, the word ‘protein’ appeared as a very frequent word.

**Figure 3:**
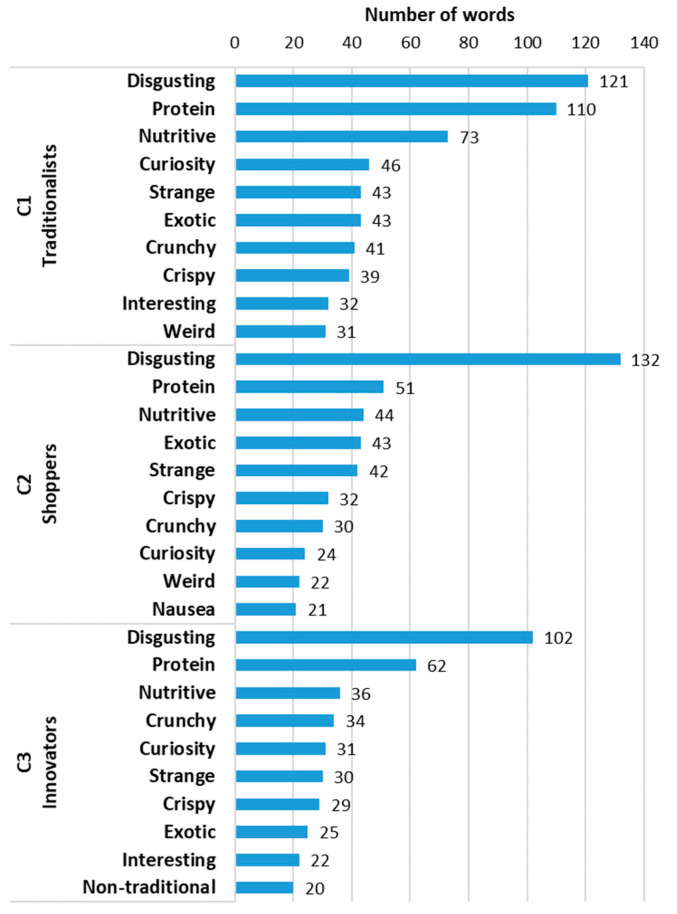
Top ten most frequent words mentioned in each cluster.

The graph in Figure 3 shows, for each cluster, the ten most frequent words for easier comparison. The results indicate that the three most frequent words were the same in all three clusters (disgusting, protein, and nutritive), although with different numbers of occurrences. These words are indicative of neophobia and rejection, but also the recognition of the nutritive value of EIs, and particularly their richness in protein. At the level of the fourth word, some differences appeared, with the word ‘curiosity’ for cluster C1, ‘exotic’ for cluster C2 and ‘crunchy’ for cluster C3. The fifth word was ‘strange’ for clusters C1 and C2 and ‘curiosity’ for cluster C3, being close to the fourth position in cluster C1. Words such as ‘crunchy’ and ‘crispy’ also appeared in the top 10 of all three clusters. These words relate to the sensorial appeal of EIs, while also expressing feelings of curiosity and the recognition of its exotic nature.

## 4. Discussion

Table 4 identified which survey items reliably measure perceptions about EIs. The factorial analysis clarifies item groupings, reveals weak or inconsistent items, and strengthens construct validity [39]. By removing low-loading items and refining factors, this study improves the survey’s precision, coherence, and ability to capture culturally diverse attitudes towards EIs. The factor analysis yielded a robust six-dimensional structure, i.e., Gastronomy, Social Aspects and Income, Tradition, Motivations, Health Risks, and Purchasing, derived from 24 items with acceptable to excellent internal consistency (Table 5). These factors reveal the multidimensional nature of attitudes towards EIs and clarify the interplay among cultural, economic, and cognitive drivers that influence acceptance or rejection, as noted in previous studies [5,40].

The Gastronomy factor (F1) emerged as the component with the highest internal consistency and the strongest loadings, particularly for items related to culinary innovation and chef endorsement. This underscores that gastronomic framing is becoming an increasingly relevant lens for normalising EIs, in line with trends reported in other Western contexts, where insect consumption is often introduced through gourmet experiences [41]. The presence of items linking EIs to culinary education further suggests that exposure through structured learning environments may reduce neophobia over time. The Social Aspects and Income factor (F2) highlights perceptions of EIs as a livelihood and an accessible source of protein, echoing global data showing that insect farming contributes to food security and economic resilience in low-income regions. For example, Ishara et al. [42] suggested that the production and consumption of EIs in African regions could be considered an opportunity to reduce food insecurity in the following decade due to climate change. Interestingly, this factor exhibited smaller contributions to cluster differentiation, suggesting that although participants recognise the socio-economic role of EIs, these considerations do not strongly drive personal acceptance or purchasing intentions.

On the other hand, the Tradition factor (F3) captures beliefs about cultural embeddedness and the influence of Westernisation, as well as perceived seasonal and religious associations. These findings emphasise the strong cultural anchoring of EI consumption and align with the literature, which shows greater acceptance in regions where entomophagy is historically rooted [43]. However, even in those countries, certain factors influence consumption across regions due to the erosion of gastronomic traditions and variations in income levels [44]. Regarding the Motivations factor (F4), it demonstrates that both cognitive and market-related determinants shape acceptance. Notably, knowledge emerged as a key driver, aligning with the prior evidence showing that consumer education increases the willingness to try EIs. Again, reducing food neophobia (F1) has a strong effect on the F4 factor, increasing EI consumption individually and collectively, as previously reported by Kiumba et al. [45].

Finally, the Health Risks factor (F5) and Purchasing factor (F6) confirm that concerns about safety and availability remain substantial barriers. High loadings for health-risk items reflect persistent concerns about contamination and hygiene, consistent with widespread misconceptions reported in previous studies [46,47]. The clusters obtained (Traditionalists, Shoppers, and Innovators) were meaningfully differentiated by the factors in Table 5, reinforcing the validity of the scale and confirming that perceptions were not homogeneous.

The results presented in Table 6 indicate that all six extracted factors were statistically significant across the three analytical methods (F1–F6, all *p* < 0.001 for BGL, NN, and CE), confirming that the scale effectively discriminates among respondents with distinct perceptual profiles. This finding validates the internal structure of the instrument and reinforces the notion that perceptions towards EIs cannot be reduced to a simple dichotomy of acceptance or rejection but rather reflect a multidimensional construct. This complexity is consistent with previous research, which suggests that consumer perception of EIs is shaped by an interplay of cultural, psychological, sociodemographic, production-related, and informational factors [7,12,14].

Within this framework, C1 exhibited consistently positive scores on tradition-related values (F3), as well as on motivation and trust constructs (F4 and F5), alongside negative scores for perceived novelty (F6). These results suggest that, for this segment, EIs are not viewed as an innovation but rather as symbols of continuity, cultural legitimacy, and reliability. In societies where EIs are culturally embedded, such perceptions strongly predict favourable acceptance. Conversely, in Western societies, where entomophagy lacks cultural grounding, cultural dissonance emerges as a substantial barrier to acceptance [8].

A contrasting pattern was observed in C3, which demonstrated the strongest positive associations with innovation-oriented dimensions (F1 and F6) and the most pronounced negative scores for tradition (F3). This inverse relationship supports the hypothesis that geographical and demographic contexts play a decisive role in shaping perception profiles. In this sense, insects are constructed as traditional food resources or as novel products, depending largely on the cultural background. This interpretation is supported by evidence showing that, in regions such as Asia, Africa, and parts of Latin America, insects are commonly recognised as traditional foods, whereas in Western countries, they are predominantly regarded as novel or alternative food sources [2,48]. Furthermore, demographic trends reinforce this pattern, as Rehman and Ogrinc [10] reported that younger individuals, those with higher educational levels, and men are more likely to exhibit openness towards EI consumption, positioning them as priority segments for targeted promotional strategies.

Finally, C2 presented weak or negative loadings on motivation and trust factors (F4 and F5), reflecting lower attitudinal engagement and a predominantly utilitarian orientation towards food. For this segment, EIs are not associated with symbolic, cultural, or emotional value, but are instead evaluated as market commodities. Their purchase decision relies primarily on practical considerations such as price, availability, and product functionality, including form and convenience, as a mechanism to offset limited attitudinal readiness [10]. This finding underscores that acceptance within this group is contingent upon tangible market conditions rather than cultural integration or innovation appeal.

In studies on food perception, sociodemographic data allow researchers to segment respondents effectively. Variables such as those listed in Table 3 often influence individuals’ acceptance levels, purchasing preferences, interests, needs, and price sensitivity. These data help identify which groups show greater interest in or acceptance of specific foods [49]. For instance, they can reveal whether a food product is better valued by people with higher education levels, whether younger consumers show greater purchase intention, whether sex influences product preference, or whether regional differences within a country affect habitual food consumption. Table 7 shows significant associations between clusters and sociodemographic variables. Regarding sex, females and males showed a similar distribution across clusters, whereas individuals identified as ‘other’ were more frequently classified in C3. These differences have been linked to psychological traits such as disgust sensitivity and novelty seeking. Men tend to exhibit lower aversion and higher sensation-seeking tendencies, while women often require greater reassurance or concealment of insect origins [13].

Although the age group was significantly associated with cluster distribution, in all groups, the majority of the participants were in cluster C1. In contrast, for the group of mature adults (31-50 years), the lowest representation was for cluster C2 (28.0%), contrary to the other two age groups, whose lowest representation was in C3. Younger individuals are primarily driven by protein content and curiosity, whereas middle-aged adults show greater concern for sustainability and cost [12,14].

Educational level influenced perception profiles. Although for all groups the highest representation was in C1, differences were registered regarding the lowest representation, with those with a university degree being less frequently classified in C2 (25.7%), whereas those in the other two groups were less associated with C3. Higher education in Western contexts has been reported as a predictor of acceptance [10], and in Romania, education level was correlated with knowledge of insects as a sustainable food source [11].

Country of residence showed the strongest association with cluster membership. Lebanon and Mexico had the highest proportions of people in C1 (73.1% and 60.1%), Lithuania in C3 (50.4%), and Croatia and Portugal mainly in C2 (45.9% and 40.6%). In Mexico, postgraduate respondents reported marketing factors (availability, price, and variety) as the main motivators, and familiarity with nutritional content also influenced interest [44]. The living environment showed no association (*p* = 0.071).

Family income was also associated with cluster distribution. Lower-income groups were mainly in C1, whereas C3 was more frequent among participants with the highest level of income (27.0%). The professional area showed significant differences only in the cases of Agriculture, Biology, Health, and other fields. For Agriculture and Biology, the participants showed a lower representation in C2 (28.4% and 23.3%, respectively), and for Health professionals, the lowest representation was in C3 (25.0%).

Regarding the results presented in Table 8, the segmentation analysis revealed important differences among consumers’ motivations and behavioural patterns towards EI consumption. The C1 exhibited the greatest experience with EI consumption and the highest willingness to consume both whole insects and insect-derived ingredients. This cluster perceives EIs through a hedonic–functional lens, which aligns with previous studies showing that sensory familiarity and nutritional benefits are key determinants in the adoption of entomophagy [50]. In contrast, EI consumption among C2 is more situational. Participants in this group reported that they would consume EIs primarily in contexts of food scarcity or environmental concern, indicating that their acceptance depends on external factors. The moderate consumption frequency observed in C2 suggests that EI consumption is an adaptive or context-dependent choice. This is consistent with research indicating that pragmatic motivations such as sustainability or necessity may promote acceptance without necessarily fostering long-term habitual adoption [51,52]. C3 showed an attitudinal profile characterised by endorsement of environmental motivations. This trend has been widely documented in studies on sustainable food consumption, which demonstrate that pro-environmental intentions are often insufficient to overcome behavioural barriers such as a lack of familiarity, limited product availability, or residual neophobic responses [53,54]. In summary, given the heterogeneity of consumer orientations, it is essential to tailor marketing strategies for the consumption of whole insects or insect-based products. For C1, it is essential to highlight the sensory quality and culinary versatility of EIs. To strengthen acceptance among C2, highlighting the sustainability and resilience benefits associated with EI consumption could be effective. Finally, for C3, efforts should focus on reducing the attitude–behaviour gap through strategies such as culinary demonstrations, tasting experiences, and pairing insects with other foods.

Participants from all clusters mentioned ‘disgusting’ as the word most strongly associated with EIs (Figure 2), even within C1, which included participants from all countries. Insect consumption is strongly linked to gastronomic tradition in many parts of the world; among the countries included in this study, only Mexico has such a tradition, whereas in European societies, this practice is often associated with food aversion [44,55]. Although the most frequently mentioned word across all clusters carries a negative meaning, the second and third most common terms were ‘protein’ and ‘nutritive’ (Figure 2 and Figure 3), which denote perceived health benefits associated with consuming insects. In a study conducted in Mexico, Portugal, Croatia, Romania, and Lithuania, focusing on individuals who had never consumed insects in their lives, the words ‘protein’ and ‘nutrition’ ranked among the ten most mentioned by participants in all countries except Croatia [56]. This indicates awareness of the nutritional value of insects, even among people who express aversion to this type of food.

A high degree of similarity was observed in the top ten words mentioned across all clusters (Figure 3). The words ‘crunchy’ and ‘crispy’, cited by all clusters, may be related to the texture of insects when consumed whole—something that, in general, is not well liked by consumers [57]. Other terms mentioned in all clusters included ‘exotic’, ‘strange’, ‘weird’, ‘curiosity’, and ‘interesting’. These terms may reflect unfamiliarity or limited availability of insects as food or an interest in trying innovative foods with high nutritional value. In line with this, it may be advisable to incorporate EIs as ingredients in food formulations, integrate insects into each country’s traditional cuisine, inform the public about their nutritional value, and develop production strategies to ensure the availability of insects at low prices [56,58].

## 5. Conclusions

This study allowed validating, through exploratory factor analysis, a scale for perceptions regarding EIs, resulting in a set of 24 items distributed by six factors: F1—Gastronomy; F2—Social Aspects and Income; F3—Tradition; F4—Motivations; F5—Health risks; and finally F6—Purchasing. Furthermore, these perceptions were the basis for cluster analysis, which allowed discriminating the participants according to their perceptions about EIs in three clusters: C1—Traditionalists; C2—Shoppers; and C3—Innovators. Statistical analysis revealed significant differences between the clusters for the sociodemographic variables country, sex, education, living environment, and income. Also, significant differences were observed for the variables related to the consumption of EIs.

A word analysis was performed based on the clusters obtained, resulting in a total of 2263 validated words. The three most frequent words were ‘disgusting’, ‘protein’, and ‘nutritive’, a trend common to all three clusters. Differences between the clusters were observed in the fourth most frequent word, which was ‘curiosity’ for cluster C1, ‘exotic’ for cluster C2, and ‘crunchy’ for cluster C3.

This work showed valuable insights into the differences in the perceptions about EIs according to country of origin and sociodemographic groups, as well as according to past experience regarding the consumption of EIs or the motivations for their consumption in the future.

Since EIs have been suggested as a more sustainable source of animal protein when compared to other meat sources, it is relevant to understand the perceptions of the individuals and in what way those perceptions can be modelled towards a high knowledge about the advantages associated with the consumption of EIs, not only as a nutritive and safe food, but also as an environmentally friendly food with a high gastronomic potential.

## 6. Implications

The findings from this work have direct and indirect implications for stakeholders along the value chain, such as producers, retailers and consumers. At the first level, we identify some implications for producers, including farmers, food processors and product developers in three major areas: (a) Product development and innovation, like developing familiar formats to reduce the ‘disgust’ barrier identified in the word analysis, or segmenting product design according to the clusters identified: for Traditionalists—integrate insects into traditional dishes or local gastronomy; for Shoppers—emphasise convenience and product availability; and for Innovators—offer novel textures and culinary experiences; (b) communication of benefits, highlighting the nutritional value (for example, protein and other nutrients) since these were highly frequent positive associations, emphasizing environmental sustainability compared with conventional livestock, as well as addressing health risk concerns by providing clear safety certifications, transparent production standards, and traceability; and (c) local economic development, using the Social Aspects and Income factor to promote rural entrepreneurship, small-scale insect farming and income diversification for farmers.

At the second level, we identified some implications for Retailers and Food Services (supermarkets, restaurants, or online platforms): (a) market segmentation strategies, using the cluster profiles for targeted marketing: for Traditionalists—introduce EIs in traditional cuisine contexts, incorporate insects in familiar dishes in restaurants (e.g., sauces, fillings), and highlight the aspects linked to heritage, gastronomy, and culinary curiosity; for Shoppers—position products as exotic or novel foods, use a retail strategy focusing on eye-catching packaging, tasting events, and promotional displays; and finally for the last cluster Innovators—focus on novelty and sensory appeal and promote crunchy snacks, gourmet dishes, and innovative recipes; (b) consumer education in retail, by providing in-store information panels explaining the nutritional benefits, sustainability advantages, and safe consumption; and (c) sampling and experience, offering tasting events, which can reduce disgust and increase curiosity, or use gastronomic storytelling to enhance perceived value.

On the third level, some implications for Consumers were also identified: (a) increasing acceptance, by encouraging gradual exposure, starting with processed products (flour-based foods) and progressing to more visible forms, and also by promoting culinary experimentation, especially for consumers with curiosity about novel foods; and (b) health and sustainability awareness, through the dissemination of accessible information on protein quality, environmental benefits compared with traditional meat and food safety regulations.

On the fourth level, some implications for Policy Makers and Food System Actors were identified: (a) education and awareness campaigns, through public campaigns highlighting the nutritional benefits, low environmental impact and the food safety standards; (b) regulatory support, by developing clear labelling regulations and quality standards for edible insect products; and (c) research and innovation support, through funded research on product development, consumer acceptance and sustainable insect farming technologies.

Finally, at the level of cross-value chain strategies, it is important to address the disgust barrier, since ‘disgusting’ was the most frequent association, and stakeholders should normalise consumption through culinary integration and emphasise taste, texture, and nutrition, while also reducing the visual exposure of whole insects. It is also relevant to invest in tailored communication, since different messages should be used depending on the consumer segment: focusing on health and protein when targeting the general population, gastronomy and tradition when targeting the Traditionalists, and novelty and texture when targeting the Innovators.

## 7. Limitations and Suggestions for Future Work

Some limitations were identified related to the sample used. One of the limitations relates to the selection of countries. We tried to include countries from a wide diversity of geographical locations, namely, we included Mexico (in Latin America), Portugal (in the Iberian Peninsula), Lithuania (in Northern Europe), Croatia (in the South European Mediterranean Coast), Romania (in Central Europe) and Lebanon (in the Middle East Mediterranean Coast). Nevertheless, in most of these countries, there is no reported typical use of EIs, except in Mexico. Therefore, we tried to use the example of Mexico in Latin America as a way to compare with other geographies in Europe and around the Mediterranean. We understand that the use of these countries certainly limited the conclusions, but for future work, it is suggested to extend this work to more countries in Africa and in Asia, where a tradition of consuming insects might be present. Future work on a higher number of countries with a wider geographical representation, especially including countries with a tradition of entomophagy, is suggested for the continuation of this exploratory work.

Another limitation results from this work being conducted on a convenience sample, which has the following limitations: (a) Limited Representativeness: since participants are not randomly selected, the sample may not accurately represent the target population, certain groups may be overrepresented or underrepresented, and this was visible in the different groups of age, sex, education, and other sociodemographic variables. (b) Reduced Generalisation: because of the lack of representativeness, the findings cannot be confidently generalised to the entire population, and should be limited to the study sample. (c) Sampling Bias: convenience samples often introduce selection bias because individuals who are easily accessible or willing to participate may share similar characteristics, attitudes, or behaviours. (d) Potential Sociodemographic Imbalances: variables such as age, education, income, and living environment may be unevenly distributed, which can influence responses and affect the interpretation of results. (e) Risk of Self-Selection Bias: participants who choose to respond may have stronger opinions or greater interest in the topic, potentially skewing results. Nevertheless, the convenience samples also have many advantages, like facilitating the data collection, especially allowing for the collection of data from participants who are readily accessible, and making the process straightforward and practical, which is especially useful when it is difficult to reach out to a broader population. Other advantages are low cost and time efficiency; since participants are easily available, convenience samples typically require less time, fewer resources, and lower financial costs when compared with probability sampling methods. Also, the convenience samples show feasibility for exploratory or preliminary research, where the main aim is to identify patterns, test instruments (e.g., questionnaire scales), or generate hypotheses rather than produce highly generalizable results. In fact, one of the areas where convenience sampling is particularly useful is for scale development, when the objective includes testing or validating measurement scales (e.g., through exploratory factor analysis, which was the present case), because convenience samples can still provide valuable information about the structure and reliability of constructs.

Based on the suitability of the use of convenience samples to conduct the validation of measurement scales through exploratory factor analysis, we suggest that future work should conduct a confirmatory factor analysis through Structural Equation Modelling, which could be an interesting approach to extend the present research.

## Figures and Tables

**Table 1:** Items utilised to measure perceptions about EIs.

N°	List of Items
1	Insects are considered a traditional food in my country.
2	Consuming insects is characteristic of developing countries.
3	Insects are present in events related to religious rituals.
4	Insects are part of the gastronomic culture of most countries in the world.
5	In some countries, the tradition of eating insects is decreasing because of the “Westernisation” of diets.
6	Insect consumption is seasonal, so it varies according to the time of the year.
7	There are obstacles to consumers’ acceptance of edible insects in Western countries.
8	Insects can be associated with traditional festivities and celebrations.
9	Insects are considered exotic foods.
10	Insects are traded as treats/delicacies.
11	Insects are associated with taboos and food neophobia (not wanting to eat unfamiliar foods).
12	Some gourmet restaurants use edible insects in their culinary preparations.
13	Insects are present in culinary events and gastronomic shows.
14	Insects are recommended by some recognised chefs.
15	Chefs contribute to the popularisation of insects in gastronomy in Western countries.
16	Culinary education favours an overall liking for innovative insect-based products.
17	Insect production can contribute to increasing the income of families in low-income areas.
18	Insects provide protein foods at low prices.
19	The market for edible insects is expected to decline in the future.
20	Presently, the Asia-Pacific and Latin America areas account for more than half of the edible insects market.
21	In some countries, insect farming is becoming a key factor in fighting against rural poverty.
22	The income generated from insects can be affected by market fluctuations in price derived from availability.
23	Edible insects are difficult to find for sale on street markets.
24	Edible insects are easy to find for sale in supermarkets.
25	Edible insects are on sale only in specialised shops.
26	The level of knowledge influences the willingness to purchase insect food.
27	Price is among the motivations to consume insect foods.
28	The consumption of insects and derived foods depends on availability.
29	Personalities/influencers can lead people to consume insects.
30	Insect consumption is independent of marketing campaigns.
31	Insects are used by some people in traditional medicine.
32	Eating insects poses a substantial risk to human health.
33	Industrial processed insect products are hygienic and safe.
34	Insects and insect-based foods are often infected by pathogens and parasites.
35	Insects collected from the wild may be contaminated with pesticide residues.
36	In certain countries, insects are approved officially for therapeutic treatment.

**Table 2:** Similarity between solutions obtained by different hierarchical clustering methods.

Methods ^1^	BGL	WGL	NN	FN	CE	WA
BGL	100%					
WGL	46%	100%				
NN	100%	46%	100%			
FN	54%	49%	54%	100%		
CE	100%	46%	100%	54%	100%	
WA	44%	32%	44%	55%	53%	100%

^1^ BGL = Between-Groups Linkage; WGL = Within-Groups Linkage; NN = Nearest Neighbour; FN = Furthest Neighbour; CE = Centroid; WA = Ward.

**Table 3:** Sociodemographic distribution of the participants.

Variables and Groups	Global Sample (N = 3711; 100%)	Croatia (n = 686; 18.5%)	Lebanon (n = 357; 9.6%)	Lithuania (n = 510; 13.7%)	Mexico (n = 1139; 30.7%)	Portugal (n = 527; 14.2%)	Romania (n = 492; 13.3%)
	% of Participants for Each Country in Each of the Groups						
Age ^1^	*p* < 0.001; V = 0.252						
Young adults (18–30 y)	50.0	52.0	41.4	56.0	53.9	63.7	19.8
Mature adults (31–50 y)	35.9	23.5	35.6	26.4	38.1	33.2	60.9
Senior adults (51+ y)	14.1	24.5	23.0	17.6	8.0	3.1	19.3
Gender ^1^	*p* < 0.001; V = 0.228						
Female	57.0	73.0	54.8	42.6	54.5	43.6	87.2
Male	42.2	26.6	44.8	54.3	44.1	56.0	12.8
Did not want to report	0.8	0.4	0.4	3.1	1.4	0.4	0.0
Education ^1^	*p* < 0.001; V = 0.335						
Under-university	39.0	46.3	65.5	4.8	31.2	45.6	12.2
University degree	37.8	21.8	31.6	58.8	38.6	42.1	37.2
Post-graduate (MSc or PhD)	23.2	31.9	2.9	36.4	30.2	12.3	50.6
Living environment ^1^	*p* < 0.001; V = 0.231						
Rural	14.2	31.3	19.7	21.3	4.5	6.3	11.8
Urban	68.8	56.2	61.9	56.9	63.9	78.5	83.1
Suburban	17.0	12.5	18.4	21.8	31.6	15.2	5.1
Family income ^1^	*p* < 0.001; V = 0.195						
Much below average	7.5	8.2	1.9	3.6	15.8	1.0	7.5
Below average	17.9	15.8	14.5	15.4	28.9	9.1	17.9
Average	36.8	38.4	55.8	44.8	20.4	31.9	36.8
Above average	30.2	26.6	24.9	29.1	28.0	48.4	30.2
Much above average	7.6	11.0	2.9	7.0	7.0	9.6	7.6

^1^ Chi-square tests to assess differences between countries for each of the sociodemographic variables: *p* = significance (*p* < 0.05), and V = Cramer’s coefficient.

**Table 4:** Initial solution obtained through FA.

Factor	%VE ^1^	Items	Loadings	Cronbach’s Alpha (α)
F1	10.65	10. Insects are traded as treats/delicacies	0.522	0.846 0.858 ^2^
		12. Some gourmet restaurants use edible insects in their culinary preparations	0.728	
		13. Insects are present in culinary events and gastronomic shows	0.763	
		14. Insects are recommended by some recognised chefs	0.779	
		15. Chefs contribute to the popularisation of insects in gastronomy in Western countries	0.678	
		16. Culinary education favours overall liking for innovative insect-based products	0.597	
F2	9.02	17. Insect production can contribute to increasing the income of families in low-income areas	0.641	0.780 ^3^
		18. Insects provide protein foods at low prices	0.674	
		20. Presently, the Asia-Pacific and Latin America areas account for more than half of the edible insects market	0.594	
		21. In some countries, insect farming is becoming a key factor in fighting against rural poverty	0.743	
		22. The income generated from insects can be affected by market fluctuations in price derived from availability	0.545	
F3	7.74	2. Consuming insects is characteristic of developing countries	0.547	0.738 ^3^
		3. Insects are present in events related to religious rituals	0.661	
		4. Insects are part of the gastronomic culture of most countries in the world	0.575	
		5. In some countries, the tradition of eating insects is decreasing because of the “Westernisation” of diets	0.543	
		6. Insect consumption is seasonal, so it varies according to the time of the year	0.539	
		7. There are obstacles to consumers’ acceptance of edible insects in Western countries	0.417	
		8. Insects can be associated with traditional festivities and celebrations	0.611	
F4	6.10	26. The level of knowledge influences the willingness to purchase insect food	0.555	0.695 ^3^
		27. Price is among the motivations to consume insect foods	0.632	
		28. The consumption of insects and derived foods depends on availability	0.674	
		29. Personalities/influencers can lead people to consume insects	0.516	
F5	5.19	32. Eating insects poses a substantial risk to human health	0.718	0.678 ^4^
		34. Insects and insect-based foods are often infected by pathogens and parasites	0.760	
F6	5.18	9. Insects are considered exotic food	0.495	0.431 ^5^
		11. Insects are associated with taboos and food neophobia (not wanting to eat unfamiliar foods)	0.569	
		35. Insects collected from the wild may be contaminated with pesticide residues	0.617	
F7	4.80	1. Insects are considered a traditional food in my country	−0.407	0.095 0.577 ^6^
		23. Edible insects are difficult to find for sale on street markets	0.781	
		24. Edible insects are easy to find on sale in supermarkets	−0.432	
		25. Edible insects are on sale only in specialised shops	0.695	
F8	3.82	19. The market of edible insects is expected to decline in the future	0.677	0.228 ^7^
		30. Insect consumption is independent of marketing campaigns	0.618	

^1^ VE = variance explained. ^2^ Alpha if item 10 is removed. ^3^ The removal of any item does not improve the value of alpha. ^4^ The factor F6 includes only two items, so it is not possible to test the removal of any item. ^5^ Unacceptable value of alpha. ^6^ Alpha if item 1 is removed from the factor. ^7^ Alpha if item 24 is removed from the factor.

**Table 5:** Final solution obtained through FA, restricting the items to the 24 items considered viable.

Factor	%VE ^1^	Items	Loadings	Factor Name	Cronbach’s Alpha (α)
F1	13.42	12. Some gourmet restaurants use edible insects in their culinary preparations	0.718	Gastronomy (GA)	0.856
		13. Insects are present in culinary events and gastronomic shows	0.780		
		14. Insects are recommended by some recognised chefs	0.815		
		15. Chefs contribute to the popularisation of insects in gastronomy in Western countries	0.735		
		16. Culinary education favours overall liking for innovative insect-based products	0.640		
F2	11.06	17. Insect production can contribute to increasing the income of families in low-income areas	0.652	Social Aspects and Income (SI)	0.780
		18. Insects provide protein foods at cheap prices	0.668		
		20. Presently, the Asia-Pacific and Latin America areas account for more than half of the edible insects market	0.651		
		21. In some countries, insect farming is becoming a key factor in fighting against rural poverty	0.733		
		22. The income generated from insects can be affected by market fluctuations in price derived from availability	0.598		
F3	10.21	3. Insects are present in events related to religious rituals	0.511	Tradition (TR)	0.724
		4. Insects are part of the gastronomic culture of most countries in the world	0.485		
		5. In some countries, the tradition of eating insects is decreasing because of the “Westernisation” of diets	0.659		
		6. Insect consumption is seasonal, so it varies according to the time of the year	0.673		
		7. There are obstacles to consumers’ acceptance of edible insects in Western countries	0.578		
		8. Insects can be associated with traditional festivities and celebrations	0.615		
F4	8.32	26. The level of knowledge influences the willingness to purchase insect food	0.527	Motivations (MT)	0.695
		27. Price is among the motivations to consume insect foods	0.714		
		28. The consumption of insects and derived foods depends on availability	0.716		
		29. Personalities/influencers can lead people to consume insects	0.547		
F5	6.72	32. Eating insects poses a substantial risk to human health	0.763	Health Risks (HR)	0.678
		34. Insects and insect-based foods are often infected by pathogens and parasites	0.801		
F6	6.41	23. Edible insects are difficult to find for sale on street markets	0.766	Purchasing (PC)	0.577
		25. Edible insects are on sale only in specialised shops	0.762		

^1^ VE = variance explained.

**Table 6:** Results for the k-means clustering, with three initial solutions.

Initial Solution ^1^	Factors	ANOVA		Cluster 1 Traditionalists		Cluster 2 Shoppers		Cluster 3 Innovators	
		F	*p*-Value	PC ^2^	FCC ^3^	PC ^2^	FCC ^3^	PC ^2^	FCC ^3^
BGL	F1 (GA)	2014	*p* < 0.001	43%	0.220	27%	−1.124	30%	0.720
	F2 (SI)	41	*p* < 0.001		−0.127		−0.041		0.221
	F3 (TR)	868	*p* < 0.001		0.599		−0.142		−0.733
	F4 (MT)	142	*p* < 0.001		0.306		−0.277		−0.186
	F5 (HR)	196	*p* < 0.001		0.357		−0.264		−0.272
	F6 (PC)	134	*p* < 0.001		−0.290		0.128		0.301
NN	F1 (GA)	1759	*p* < 0.001	42%	0.215	32%	0.641	26%	−1.140
	F2 (SI)	42	*p* < 0.001		0.086		0.091		−0.252
	F3 (TR)	468	*p* < 0.001		0.523		−0.444		−0.303
	F4 (MT)	285	*p* < 0.001		0.338		−0.511		0.082
	F5 (HR)	176	*p* < 0.001		0.345		−0.226		−0.281
	F6 (PC)	410	*p* < 0.001		−0.470		0.513		0.131
CE	F1 (GA)	590	*p* < 0.001	43%	0.195	29%	−0.751	28%	0.470
	F2 (SI)	40	*p* < 0.001		−0.017		0.204		−0.181
	F3 (TR)	1447	*p* < 0.001		0.594		0.098		−1.005
	F4 (MT)	402	*p* < 0.001		0.261		−0.664		0.281
	F5 (HR)	153	*p* < 0.001		0.294		−0.360		−0.079
	F6 (PC)	304	*p* < 0.001		−0.424		0.414		0.223

^1^ BLG = Between-Groups Linkage; NN = Nearest Neighbour; CE = Centroid; ^2^ PC = percentage of cases in the cluster. ^3^ FCC = final cluster centres.

**Table 7:** Cluster membership according to sociodemographic characteristics.

Variables	Groups	C1 Traditionalists	C2 Shoppers	C3 Innovators	Total
Sex (*p* = 0.017; V = 0.040)	Female	42.3%	28.2%	29.5%	100%
	Male	43.9%	30.0%	26.1%	100%
	Other	40.7%	11.1%	48.1%	100%
Age group (*p* < 0.001; V = 0.063)	Young adults (18–30 y)	46.6%	27.6%	25.8%	100%
	Mature adults (31–50 y)	40.9%	28.0%	31.0%	100%
	Senior adults (51 y or over)	36.3%	35.3%	28.4%	100%
Education level (*p* = 0.003; V = 0.047)	Postgraduate education (MSc or PhD)	43.5%	28.7%	27.8%	100%
	University degree	46.2%	25.7%	28.1%	100%
	No university degree	39.7%	31.8%	28.5%	100%
Country (*p* < 0.001; V = 0.290)	Croatia	24.4%	45.9%	29.7%	100%
	Lebanon	73.1%	10.4%	16.5%	100%
	Lithuania	17.5%	32.2%	50.4%	100%
	Mexico	60.1%	17.9%	21.9%	100%
	Portugal	29.2%	40.6%	30.2%	100%
	Romania	48.0%	28.8%	28.2%	100%
Living environment (*p* = 0.071; V = 0.034)	Rural	47.1%	29.7%	23.3%	100%
	Urban	42.0%	29.0%	29.0%	100%
	Suburban	43.4%	27.5%	29.1%	100%
Family income (*p* < 0.001; V = 0.100)	Much below average	58.1%	17.3%	24.5%	100%
	Below average	51.1%	27.5%	21.4%	100%
	Average	39.1%	31.2%	29.8%	100%
	Above average	41.1%	27.1%	31.8%	100%
	Much above average	37.1%	36.0%	27.0%	100%
Professional area *	Food/Nutrition	44.7%	25.8%	29.5%	100%
	Agriculture *	39.9%	28.4%	31.8%	100%
	Environment	49.0%	23.8%	27.2%	100%
	Biology *	51.2%	23.3%	25.5%	100%
	Health *	48.2%	26.8%	25.0%	100%
	Tourism	40.8%	30.8%	28.3%	100%
	Others *	43.3%	29.9%	26.7%	100%

* Each option was a yes/no question, and significant differences were observed according to the Chi-square test only for professional areas of Agriculture (*p* = 0.022; V = 0.048), Biology (*p* = 0.001; V = 0.065), Health (*p* = 0.011; V = 0.052), and Others (*p* = 0.025; V = 0.047).

**Table 8:** Cluster membership according to consumption habits related to EIs.

Variables	Groups	C1 Traditionalists	C2 Shoppers	C3 Innovators	Total
Have you consumed EIs or derived foods? (*p* < 0.001; V = 0.112)	Yes	54.1%	20.1%	25.8%	100%
	No	37.8%	32.9%	29.4%	100%
	Do not know/do not remember	48.3%	25.0%	26.7%	100%
If you have not consumed EIs, would you consider consuming? (*p* < 0.001; V = 0.097)	Definitely not	37.2%	35.1%	27.7%	100%
	Maybe	38.3%	28.6%	33.1%	100%
	Yes, but only derived foods that include insects (for example, hamburger or biscuits)	47.6%	23.9%	28.5%	100%
	Yes, whole insects and derived foods	52.0%	22.3%	25.6%	100%
In which circumstances would you consume EIs? *	Out of curiosity *	43.4%	27.6%	28.9%	100%
	If there is a scarcity of food *	38.9%	33.8%	27.4%	100%
	To help preserve the planet	36.3%	30.3%	33.4%	100%
	Because of the gastronomic characteristics	53.0%	21.8%	25.3%	100%
	Because of the nutritional properties	49.3%	22.5%	28.2%	100%
If you consume EIs, how often? (*p* = 0.010; V = 0.081)	About one time per year	54.2%	21.3%	24.5%	100%
	About two–three times per year	65.4%	14.4%	20.1%	100%
	About one time per month	63.9%	13.9%	22.1%	100%
	About one time per week	70.4%	14.8%	14.8%	100%
	Two or more times per week	52.9%	29.4%	17.6%	100%

* Each option was a yes/no question, and significant differences were observed according to the Chi-square test for all options (*p* < 0.001) except for option ‘To help preserve the planet’ (*p* = 0.257). The values of Cramer’s V in order of the answering options are 0.095, 0.087, 0.039, 0.208, and 0.171.

## Data Availability

The original contributions presented in this study are included in the article. Further inquiries can be directed to the corresponding author.

## Appendix A

In Table A1, the results of the word analysis are presented in detail, with all words obtained for each of the three clusters and the corresponding number of occurrences.

**Table A1:** Word analysis according to the cluster membership.

Clusters	Number of Members	Total Number of Words	Number of Different Words	Words and Count
C1	1590	882	56	Disgusting (121), Protein (110), Nutritive (73), Curiosity (46), Strange (43), Exotic (43), Crunchy (41), Crispy (39), Interesting (32), Weird (31), Tasty (22), Inedible (22), Tradition (21), Different (18), Snack (15), Nausea (15), Delicious (15), Sustainable (12), Horrible (12), Future (10), Ugly (8), Healthy (8), Alternative (8), Unpleasant (7), New (7), Innovation (7), Filth (7), Fear (7), Dirty (7), Tasteless (5), Hunger (5), Good (5), Edible (5), Vomit (4), Unacceptable (4), Repulsion (4), Gastronomy (4), Experience (4), Bad (4), Unusual (3), Non-traditional (3), Gourmet (3), Culture (3), Unknown (2), Unattractive (2), Surprise (2), Spicy (2), Novelty (2), Nasty (2), Uncommon (1), Intriguing (1), Insecurity (1), Food (1), Exciting (1), Discomfort (1), Awful (1)
C2	1068	711	56	Disgusting(132), Protein (51), Nutritive (44), Exotic (43), Strange (42), Crispy (32), Crunchy (30), Curiosity (24), Weird (22), Nausea (21), Unusual (18), Alternative (16), Horrible (15), Inedible (14), Different (14), Repulsion (13), Non-traditional (13), Nasty (11), Dirty (11), Bad (11), Interesting (10), Tasty (9), Sustainable (9), Vomit (8), Hunger (7), Tradition (6), Delicious (6), Tasteless (5), Future (5), Unpleasant (4), Snack (4), New (4), Gastronomy (4), Foreign (4), Food (4), Uncommon (3), Unattractive (3), Healthy (3), Gourmet (3), Fear (3), Experience (3), Discomfort (3), Unknown (2), Unacceptable (2), Ugly (2), Novelty (2), Intriguing (2), Insecurity (2), Good (2), Filth (2), Edible (2), Culture (2), Unhygienic (1), Neophobia (1), Exciting (1), Awful (1)
C3	1044	670	55	Disgusting (102), Protein (62), Nutritive (36), Crunchy (34), Curiosity (31), Strange (30), Crispy (29), Exotic (25), Interesting (22), Non-traditional (20), Inedible (15), Tasteless (14), Different (14), Nausea (13), Sustainable (12), Future (12), Unattractive (11), New (11), Innovation (11), Repulsion (10), Bad (10), Alternative (10), Unusual (9), Hunger (9), Horrible (9), Healthy (9), Delicious (9), Weird (8), Vomit (8), Tasty (7), Unpleasant (6), Ugly (6), Tradition (6), Uncommon (5), Gastronomy (5), Snack (4), Gourmet (4), Fear (4), Filth (3), Culture (3), Unacceptable (2), Neophobia (2), Nasty (2), Insecurity (2), Food (2), Exciting (2), Awful (2), Unknown (1), Unhygienic (1), Surprise (1), Novelty (1), Good (1), Foreign (1), Discomfort (1), Dirty (1)
Total	3702	2263		
